# 1′-Methyl-3′-(4-methyl­benzo­yl)-4′-[5-(2-thien­yl)-2-thien­yl]spiro­[acenaphthyl­ene-1,2′-pyrrolidin]-2(1*H*)-one

**DOI:** 10.1107/S1600536810053869

**Published:** 2011-01-08

**Authors:** S. Thenmozhi, E. Govindan, D. Gavaskar, R. Raghunathan, A. SubbiahPandi

**Affiliations:** a Department of Physics, Presidency College (Autonomous), Chennai 600 005, India; b Department of Organic Chemistry, University of Madras, Guindy Campus, Chennai 600 025, India

## Abstract

In the title compound, C_32_H_25_NO_2_S_2_, the mean plane through the five-membered pyrrolidine ring, which exhibits an envelope conformation, makes dihedral angles of 82.3 (1) and 83.9 (9)° with the benzene ring and the acenaphthyl­ene ring system, respectively. The dihedral angle between the thiophene rings is 19.0(3)°. The crystal structure shows C—H⋯π and π–π inter­actions [centroid–centroid distance = 3.869 (2) Å].

## Related literature

For puckering parameters, see: Cremer & Pople (1975 ▶). For asymmetry parameters, see: Nardelli (1983 ▶).
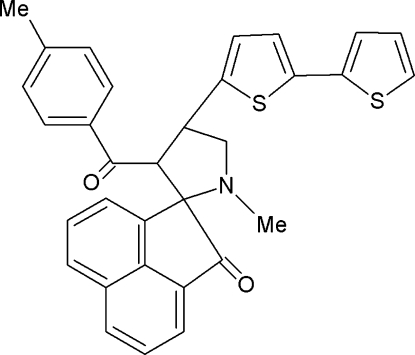


## Experimental

### 

#### Crystal data

C_32_H_25_NO_2_S_2_
*M* *_r_* = 519.65
Monoclinic, 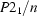

*a* = 10.2188 (5) Å
*b* = 10.0191 (5) Å
*c* = 24.8192 (11) Å
β = 96.115 (2)°
*V* = 2526.6 (2) Å^3^
*Z* = 4
Mo *K*α radiation
μ = 0.24 mm^−1^
*T* = 293 K
0.30 × 0.20 × 0.20 mm

#### Data collection

Bruker APEXII CCD area detector diffractometer
Absorption correction: multi-scan (*SADABS*; Sheldrick, 1996 ▶) *T*_min_ = 0.931, *T*_max_ = 0.953
24301 measured reflections
4373 independent reflections
3294 reflections with *I* > 2σ(*I*)
*R*_int_ = 0.041

#### Refinement

*R*[*F* ^2^ > 2σ(*F* ^2^)] = 0.040
*wR*(*F* ^2^) = 0.127
*S* = 1.06
4373 reflections
357 parameters
5 restraints
H-atom parameters constrained
Δρ_max_ = 0.31 e Å^−3^
Δρ_min_ = −0.26 e Å^−3^

### 

Data collection: *APEX2* (Bruker, 2004 ▶); cell refinement: *SAINT* (Bruker, 2004 ▶); data reduction: *SAINT*; program(s) used to solve structure: *SHELXS97* (Sheldrick, 2008 ▶); program(s) used to refine structure: *SHELXL97* (Sheldrick, 2008 ▶); molecular graphics: *ORTEP-3* (Farrugia, 1997 ▶); software used to prepare material for publication: *SHELXL97* and *PLATON* (Spek, 2009 ▶).

## Supplementary Material

Crystal structure: contains datablocks global, I. DOI: 10.1107/S1600536810053869/bt5402sup1.cif

Structure factors: contains datablocks I. DOI: 10.1107/S1600536810053869/bt5402Isup2.hkl

Additional supplementary materials:  crystallographic information; 3D view; checkCIF report

## Figures and Tables

**Table 1:** Hydrogen-bond geometry (Å, °) *Cg*8 is the centroid of the C18–C23 ring.

*D*—H⋯*A*	*D*—H	H⋯*A*	*D*⋯*A*	*D*—H⋯*A*
C32—H32⋯*Cg*8^i^	0.93	2.86	3.661 (3)	145

Symmetry code: (i) 
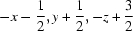
.

**Fig. 1.**
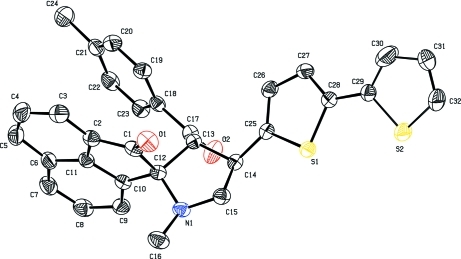
The molecular structure of the title compound showing displacement ellipsoids at the 30% probability level. For clarity, H atoms are omitted.

## supplementary crystallographic information

### Comment

Fig. 1 shows a displacement ellipsoid diagram of the molecule with the atomic numbering scheme. The geometry of the acenaphthylene moiety compares well with that reported in other compounds. The N—C and C—C bond lengths in the pyrrolidine moiety are slightly longer than the normal values reported for similar structures. This may be due to steric forces caused by the bulky substituents on the pyrrolidine moiety. The pyrrolidine ring makes dihedral angles of 82.3 (1) and 83.9 (9)° with the phenyl ring and the acenaphthylene ring system respectively. Ketone atom O1 deviates by 0.272 (2)Å from the (C1/C2/C11/C10/C12) plane. The sum of the angles at N1 of the pyrrolidine ring (340.1°) is in accordance with *sp*^3^ hybridization.

The pyrrolidine ring adopt an twist conformation, with the puckering parameters q_2_ and φ (Cremer & Pople, 1975) and the smallest displacement asymmetric parameters, Δ, (Nardelli *et al.*, 1983) as follows: q_2_= 0.379 (2) Å, φ=160.3 (3)°, Δ_s_(C12)= 16.28 (19)°. The ring (C1/C2/C11/C10/C12) adopt an twist conformation, with the puckering parameters q_2_ and φ (Cremer & Pople, 1975) and the smallest displacement asymmetric parameters, Δ, (Nardelli *et al.*, 1983) as follows: q_2_= 0.122 (2) Å, φ=162.5 (10)°, Δ_s_(C11)= 4.1 (2)°.

The molecular structure is influenced by C–H···O intramolecular interactions. In addition to van der Waals interaction, the crystal packing is stabilized by C–H···π (Table. 1) hydrogen bonds as well as by π–π electron interactions. The π–π electron interactions between the rings *Cg*7 - *Cg*8 at *x*, *y*, *z* with the centroid-centroid distance equal to 3.8655 (12) Å, is observed in the crystal structure [*Cg*7 and *Cg*8 are the centeroid of the rings C6—C11 and C18—C23].

### Experimental

A solution of the (4-chloro-phenyl-3-Bithiophenyl-prop-2-ene-1-one derived from Bithiophene (1- mmol), Acenapthoquinone (1 mmol), sarcosine (1 mmol) in toluene (30 ml) was refluxed for 8 hrs. The progress of the reacion was evidenced by the TLC analysis. The solvent was removed under reduced pressure and the crude product was subjected to column chromatogarphy using petroleum ether/ethyl acetate (4:1) as solvent. X-ray diffraction were obtained by slow evaporation of a solution of the title compound in hexene at room temperature.

### Refinement

The C and S atoms of one thiophene ring are disordered over two positions (C30/C30'and S2/S2') with refined occupancies of 0.553 (5) and 0.448 (5). The corresponding bond distances involving the disordered atoms were restrained to be equal. All H atoms were fixed geometrically and allowed to ride on their parent C atoms, with C—H distances fixed in the range 0.93–0.97 Å with *U*_iso_(H) = 1.5*U*_eq_(C) for methyl H 1.2*U*_eq_(C) for other H atoms.

### Crystal data

**Table:**

C_32_H_25_NO_2_S_2_	*F*(000) = 1088
*M**_r_* = 519.65	*D*_x_ = 1.366 Mg m^−^^3^
Monoclinic, *P*2_1_/*n*	Mo *K*α radiation, λ = 0.71073 Å
Hall symbol: -P 2yn	Cell parameters from 4373 reflections
*a* = 10.2188 (5) Å	θ = 1.6–24.9°
*b* = 10.0191 (5) Å	µ = 0.24 mm^−^^1^
*c* = 24.8192 (11) Å	*T* = 293 K
β = 96.115 (2)°	Block, colourless
*V* = 2526.6 (2) Å^3^	0.30 × 0.20 × 0.20 mm
*Z* = 4	

### Data collection

**Table:**

Bruker APEXII CCD area detector diffractometer	4373 independent reflections
Radiation source: fine-focus sealed tube	3294 reflections with *I* > 2σ(*I*)
graphite	*R*_int_ = 0.041
ω and φ scans	θ_max_ = 24.9°, θ_min_ = 1.7°
Absorption correction: multi-scan (*SADABS*; Sheldrick, 1996)	*h* = −12→12
*T*_min_ = 0.931, *T*_max_ = 0.953	*k* = −11→11
24301 measured reflections	*l* = −28→29

### Refinement

**Table:**

Refinement on *F*^2^	Secondary atom site location: difference Fourier map
Least-squares matrix: full	Hydrogen site location: inferred from neighbouring sites
*R*[*F*^2^ > 2σ(*F*^2^)] = 0.040	H-atom parameters constrained
*wR*(*F*^2^) = 0.127	*w* = 1/[σ^2^(*F*_o_^2^) + (0.0744*P*)^2^ + 0.2786*P*] where *P* = (*F*_o_^2^ + 2*F*_c_^2^)/3
*S* = 1.06	(Δ/σ)_max_ = 0.002
4373 reflections	Δρ_max_ = 0.31 e Å^−^^3^
357 parameters	Δρ_min_ = −0.26 e Å^−^^3^
5 restraints	Extinction correction: *SHELXL97* (Sheldrick, 2008), Fc^*^=kFc[1+0.001xFc^2^λ^3^/sin(2θ)]^-1/4^
Primary atom site location: structure-invariant direct methods	Extinction coefficient: 0.0070 (11)

### Special details

**Table:**

Geometry. All e.s.d.'s (except the e.s.d. in the dihedral angle between two l.s. planes) are estimated using the full covariance matrix. The cell e.s.d.'s are taken into account individually in the estimation of e.s.d.'s in distances, angles and torsion angles; correlations between e.s.d.'s in cell parameters are only used when they are defined by crystal symmetry. An approximate (isotropic) treatment of cell e.s.d.'s is used for estimating e.s.d.'s involving l.s. planes.
Refinement. Refinement of *F*^2^ against ALL reflections. The weighted *R*-factor *wR* and goodness of fit *S* are based on *F*^2^, conventional *R*-factors *R* are based on *F*, with *F* set to zero for negative *F*^2^. The threshold expression of *F*^2^ > σ(*F*^2^) is used only for calculating *R*-factors(gt) *etc*. and is not relevant to the choice of reflections for refinement. *R*-factors based on *F*^2^ are statistically about twice as large as those based on *F*, and *R*- factors based on ALL data will be even larger.

### Fractional atomic coordinates and isotropic or equivalent isotropic displacement parameters (Å^2^)

**Table:**

	*x*	*y*	*z*	*U*_iso_*/*U*_eq_	Occ. (<1)
C1	0.3304 (2)	0.1779 (2)	0.70015 (8)	0.0454 (5)	
C2	0.4379 (2)	0.1287 (2)	0.67042 (8)	0.0461 (5)	
C3	0.5595 (2)	0.0752 (2)	0.68669 (11)	0.0606 (7)	
H3	0.5867	0.0600	0.7231	0.073*	
C4	0.6408 (2)	0.0445 (3)	0.64671 (13)	0.0700 (8)	
H4	0.7224	0.0061	0.6572	0.084*	
C5	0.6064 (2)	0.0679 (3)	0.59330 (12)	0.0666 (7)	
H5	0.6654	0.0484	0.5684	0.080*	
C6	0.4809 (2)	0.1221 (2)	0.57501 (9)	0.0526 (6)	
C7	0.4291 (3)	0.1523 (3)	0.52189 (10)	0.0619 (7)	
H7	0.4794	0.1372	0.4934	0.074*	
C8	0.3062 (3)	0.2032 (2)	0.51176 (9)	0.0608 (7)	
H8	0.2749	0.2240	0.4762	0.073*	
C9	0.2238 (2)	0.2262 (2)	0.55289 (8)	0.0509 (6)	
H9	0.1395	0.2606	0.5446	0.061*	
C10	0.2702 (2)	0.1968 (2)	0.60496 (8)	0.0413 (5)	
C11	0.3991 (2)	0.1476 (2)	0.61535 (8)	0.0432 (5)	
C12	0.2087 (2)	0.2050 (2)	0.65756 (7)	0.0401 (5)	
C13	0.10321 (19)	0.0952 (2)	0.66736 (7)	0.0385 (5)	
H13	0.1418	0.0338	0.6954	0.046*	
C14	−0.0093 (2)	0.1721 (2)	0.68969 (8)	0.0410 (5)	
H14	−0.0801	0.1834	0.6602	0.049*	
C15	0.0487 (2)	0.3089 (2)	0.70413 (9)	0.0490 (5)	
H15A	−0.0190	0.3771	0.7016	0.059*	
H15B	0.0948	0.3094	0.7404	0.059*	
C16	0.2182 (3)	0.4476 (2)	0.66936 (11)	0.0677 (7)	
H16A	0.2715	0.4458	0.7036	0.102*	
H16B	0.1621	0.5246	0.6677	0.102*	
H16C	0.2740	0.4516	0.6406	0.102*	
C17	0.0632 (2)	0.0156 (2)	0.61614 (8)	0.0432 (5)	
C18	0.1546 (2)	−0.0889 (2)	0.60031 (8)	0.0412 (5)	
C19	0.2411 (2)	−0.1560 (2)	0.63759 (9)	0.0488 (5)	
H19	0.2444	−0.1342	0.6741	0.059*	
C20	0.3225 (2)	−0.2549 (2)	0.62147 (10)	0.0571 (6)	
H20	0.3790	−0.2991	0.6474	0.068*	
C21	0.3219 (2)	−0.2891 (2)	0.56801 (10)	0.0562 (6)	
C22	0.2383 (2)	−0.2199 (2)	0.53054 (9)	0.0580 (6)	
H22	0.2383	−0.2397	0.4939	0.070*	
C23	0.1546 (2)	−0.1218 (2)	0.54609 (8)	0.0507 (6)	
H23	0.0982	−0.0779	0.5201	0.061*	
C24	0.4083 (3)	−0.3996 (3)	0.55094 (13)	0.0844 (9)	
H24A	0.4895	−0.4007	0.5743	0.127*	
H24B	0.4264	−0.3850	0.5142	0.127*	
H24C	0.3642	−0.4836	0.5534	0.127*	
C25	−0.06508 (19)	0.1024 (2)	0.73551 (8)	0.0410 (5)	
C26	−0.0606 (2)	−0.0272 (2)	0.75010 (9)	0.0498 (6)	
H26	−0.0153	−0.0915	0.7324	0.060*	
C27	−0.1308 (2)	−0.0565 (2)	0.79456 (9)	0.0519 (6)	
H27	−0.1363	−0.1417	0.8089	0.062*	
C28	−0.1894 (2)	0.0515 (2)	0.81428 (8)	0.0436 (5)	
C29	−0.2731 (2)	0.0571 (2)	0.85757 (9)	0.0461 (5)	
C31	−0.3873 (3)	0.0005 (3)	0.93282 (12)	0.0756 (8)	
H31	−0.4091	−0.0482	0.9625	0.091*	
C32	−0.4376 (3)	0.1141 (3)	0.91528 (12)	0.0762 (8)	
H32	−0.5047	0.1544	0.9320	0.091*	
N1	0.13862 (18)	0.32821 (16)	0.66354 (7)	0.0466 (5)	
O1	0.33278 (17)	0.19761 (17)	0.74813 (6)	0.0642 (5)	
O2	−0.03837 (15)	0.04042 (19)	0.58800 (6)	0.0649 (5)	
S1	−0.15669 (5)	0.19129 (5)	0.77771 (2)	0.0469 (2)	
S2	−0.3801 (3)	0.1833 (4)	0.86351 (12)	0.0651 (7)	0.553 (5)
C30	−0.2931 (13)	−0.0330 (12)	0.8975 (5)	0.099 (7)	0.553 (5)
H30	−0.2465	−0.1129	0.9011	0.119*	0.553 (5)
S2'	−0.2696 (4)	−0.0663 (5)	0.90273 (15)	0.0671 (9)	0.448 (5)
C30'	−0.3667 (15)	0.1454 (14)	0.8715 (6)	0.102 (8)	0.448 (5)
H30'	−0.3827	0.2245	0.8523	0.123*	0.448 (5)

### Atomic displacement parameters (Å^2^)

**Table:**

	*U*^11^	*U*^22^	*U*^33^	*U*^12^	*U*^13^	*U*^23^
C1	0.0547 (13)	0.0417 (12)	0.0382 (12)	−0.0115 (10)	−0.0020 (10)	0.0012 (9)
C2	0.0448 (12)	0.0423 (12)	0.0493 (13)	−0.0100 (10)	−0.0034 (10)	−0.0005 (10)
C3	0.0528 (15)	0.0552 (15)	0.0691 (16)	−0.0106 (12)	−0.0150 (13)	0.0012 (12)
C4	0.0407 (14)	0.0669 (18)	0.101 (2)	−0.0018 (12)	−0.0014 (14)	−0.0031 (15)
C5	0.0465 (14)	0.0642 (17)	0.092 (2)	−0.0044 (12)	0.0177 (14)	−0.0057 (14)
C6	0.0503 (14)	0.0466 (13)	0.0632 (15)	−0.0076 (11)	0.0169 (11)	−0.0005 (11)
C7	0.0735 (18)	0.0598 (15)	0.0570 (15)	−0.0050 (13)	0.0284 (13)	0.0005 (12)
C8	0.0806 (18)	0.0639 (16)	0.0393 (12)	0.0006 (14)	0.0124 (12)	0.0099 (11)
C9	0.0566 (14)	0.0527 (13)	0.0437 (12)	0.0050 (11)	0.0065 (10)	0.0084 (10)
C10	0.0481 (12)	0.0379 (11)	0.0382 (11)	−0.0038 (9)	0.0055 (9)	0.0026 (8)
C11	0.0437 (12)	0.0376 (11)	0.0484 (12)	−0.0076 (9)	0.0060 (9)	−0.0007 (9)
C12	0.0459 (12)	0.0388 (11)	0.0350 (10)	−0.0014 (9)	0.0018 (9)	0.0001 (8)
C13	0.0418 (11)	0.0402 (11)	0.0334 (10)	0.0017 (9)	0.0033 (8)	−0.0004 (8)
C14	0.0406 (11)	0.0457 (12)	0.0365 (11)	0.0040 (9)	0.0028 (8)	−0.0024 (9)
C15	0.0595 (14)	0.0436 (13)	0.0450 (12)	0.0044 (10)	0.0107 (10)	−0.0036 (9)
C16	0.0919 (19)	0.0432 (14)	0.0706 (17)	−0.0094 (13)	0.0213 (14)	−0.0038 (12)
C17	0.0454 (12)	0.0484 (12)	0.0359 (11)	−0.0036 (10)	0.0044 (10)	−0.0033 (9)
C18	0.0473 (12)	0.0386 (11)	0.0387 (11)	−0.0092 (9)	0.0091 (9)	−0.0042 (9)
C19	0.0598 (14)	0.0408 (12)	0.0459 (12)	−0.0017 (11)	0.0053 (10)	−0.0043 (10)
C20	0.0632 (15)	0.0442 (13)	0.0640 (15)	0.0024 (11)	0.0076 (12)	−0.0001 (11)
C21	0.0611 (15)	0.0432 (13)	0.0691 (16)	−0.0049 (11)	0.0291 (12)	−0.0017 (11)
C22	0.0774 (17)	0.0545 (14)	0.0463 (13)	−0.0107 (13)	0.0268 (12)	−0.0095 (11)
C23	0.0616 (14)	0.0510 (13)	0.0408 (12)	−0.0039 (11)	0.0111 (10)	−0.0058 (10)
C24	0.094 (2)	0.0637 (17)	0.103 (2)	0.0127 (16)	0.0463 (18)	−0.0040 (16)
C25	0.0383 (11)	0.0443 (12)	0.0400 (11)	0.0029 (9)	0.0028 (9)	−0.0039 (9)
C26	0.0542 (13)	0.0460 (13)	0.0510 (13)	0.0092 (11)	0.0131 (10)	−0.0053 (10)
C27	0.0590 (14)	0.0401 (12)	0.0581 (13)	0.0053 (10)	0.0132 (11)	0.0036 (10)
C28	0.0430 (12)	0.0437 (12)	0.0438 (12)	0.0008 (9)	0.0032 (9)	−0.0019 (9)
C29	0.0441 (12)	0.0483 (13)	0.0466 (13)	0.0005 (10)	0.0079 (10)	−0.0031 (11)
C31	0.0747 (19)	0.084 (2)	0.0728 (18)	0.0009 (16)	0.0309 (15)	0.0127 (16)
C32	0.0676 (17)	0.087 (2)	0.0796 (19)	0.0142 (16)	0.0322 (15)	−0.0011 (16)
N1	0.0599 (12)	0.0349 (10)	0.0459 (10)	−0.0009 (8)	0.0104 (9)	−0.0014 (7)
O1	0.0742 (11)	0.0774 (12)	0.0387 (9)	−0.0101 (9)	−0.0044 (8)	−0.0033 (8)
O2	0.0512 (10)	0.0888 (13)	0.0518 (9)	0.0103 (9)	−0.0081 (8)	−0.0197 (8)
S1	0.0524 (4)	0.0412 (3)	0.0490 (3)	0.0046 (2)	0.0141 (3)	−0.0023 (2)
S2	0.0711 (10)	0.0674 (15)	0.0612 (11)	0.0223 (8)	0.0278 (8)	0.0127 (10)
C30	0.103 (9)	0.084 (9)	0.114 (10)	0.044 (5)	0.030 (5)	0.008 (5)
S2'	0.0722 (15)	0.0687 (19)	0.0657 (13)	0.0090 (15)	0.0328 (11)	0.0155 (11)
C30'	0.141 (14)	0.093 (13)	0.073 (7)	0.006 (8)	0.012 (7)	0.034 (6)

### Geometric parameters (Å, °)

**Table:**

C1—O1	1.205 (2)	C17—C18	1.484 (3)
C1—C2	1.472 (3)	C18—C19	1.384 (3)
C1—C12	1.567 (3)	C18—C23	1.385 (3)
C2—C3	1.375 (3)	C19—C20	1.380 (3)
C2—C11	1.395 (3)	C19—H19	0.9300
C3—C4	1.394 (4)	C20—C21	1.370 (3)
C3—H3	0.9300	C20—H20	0.9300
C4—C5	1.355 (4)	C21—C22	1.380 (3)
C4—H4	0.9300	C21—C24	1.505 (3)
C5—C6	1.421 (3)	C22—C23	1.385 (3)
C5—H5	0.9300	C22—H22	0.9300
C6—C11	1.395 (3)	C23—H23	0.9300
C6—C7	1.401 (3)	C24—H24A	0.9600
C7—C8	1.354 (3)	C24—H24B	0.9600
C7—H7	0.9300	C24—H24C	0.9600
C8—C9	1.410 (3)	C25—C26	1.347 (3)
C8—H8	0.9300	C25—S1	1.725 (2)
C9—C10	1.361 (3)	C26—C27	1.409 (3)
C9—H9	0.9300	C26—H26	0.9300
C10—C11	1.404 (3)	C27—C28	1.353 (3)
C10—C12	1.510 (3)	C27—H27	0.9300
C12—N1	1.443 (3)	C28—C29	1.444 (3)
C12—C13	1.577 (3)	C28—S1	1.721 (2)
C13—C17	1.519 (3)	C29—C30	1.371 (8)
C13—C14	1.535 (3)	C29—C30'	1.374 (9)
C13—H13	0.9800	C29—S2'	1.667 (4)
C14—C25	1.498 (3)	C29—S2	1.689 (3)
C14—C15	1.521 (3)	C31—C32	1.304 (4)
C14—H14	0.9800	C31—C30	1.411 (8)
C15—N1	1.447 (3)	C31—S2'	1.626 (5)
C15—H15A	0.9700	C31—H31	0.9300
C15—H15B	0.9700	C32—C30'	1.404 (8)
C16—N1	1.445 (3)	C32—S2	1.624 (4)
C16—H16A	0.9600	C32—H32	0.9300
C16—H16B	0.9600	C30—H30	0.9300
C16—H16C	0.9600	C30'—H30'	0.9300
C17—O2	1.213 (2)		
			
O1—C1—C2	127.8 (2)	C19—C18—C23	117.9 (2)
O1—C1—C12	124.7 (2)	C19—C18—C17	122.77 (18)
C2—C1—C12	107.48 (16)	C23—C18—C17	119.36 (19)
C3—C2—C11	119.6 (2)	C20—C19—C18	121.1 (2)
C3—C2—C1	133.1 (2)	C20—C19—H19	119.4
C11—C2—C1	107.25 (18)	C18—C19—H19	119.4
C2—C3—C4	117.7 (2)	C21—C20—C19	121.3 (2)
C2—C3—H3	121.1	C21—C20—H20	119.4
C4—C3—H3	121.1	C19—C20—H20	119.4
C5—C4—C3	123.1 (2)	C20—C21—C22	117.8 (2)
C5—C4—H4	118.5	C20—C21—C24	120.9 (3)
C3—C4—H4	118.5	C22—C21—C24	121.3 (2)
C4—C5—C6	120.7 (2)	C21—C22—C23	121.6 (2)
C4—C5—H5	119.7	C21—C22—H22	119.2
C6—C5—H5	119.7	C23—C22—H22	119.2
C11—C6—C7	116.1 (2)	C18—C23—C22	120.2 (2)
C11—C6—C5	115.5 (2)	C18—C23—H23	119.9
C7—C6—C5	128.4 (2)	C22—C23—H23	119.9
C8—C7—C6	120.4 (2)	C21—C24—H24A	109.5
C8—C7—H7	119.8	C21—C24—H24B	109.5
C6—C7—H7	119.8	H24A—C24—H24B	109.5
C7—C8—C9	122.7 (2)	C21—C24—H24C	109.5
C7—C8—H8	118.6	H24A—C24—H24C	109.5
C9—C8—H8	118.6	H24B—C24—H24C	109.5
C10—C9—C8	118.5 (2)	C26—C25—C14	130.42 (19)
C10—C9—H9	120.7	C26—C25—S1	109.82 (15)
C8—C9—H9	120.7	C14—C25—S1	119.72 (15)
C9—C10—C11	118.43 (19)	C25—C26—C27	113.94 (19)
C9—C10—C12	132.4 (2)	C25—C26—H26	123.0
C11—C10—C12	109.19 (17)	C27—C26—H26	123.0
C6—C11—C2	123.3 (2)	C28—C27—C26	113.5 (2)
C6—C11—C10	123.7 (2)	C28—C27—H27	123.3
C2—C11—C10	113.00 (19)	C26—C27—H27	123.3
N1—C12—C10	113.09 (16)	C27—C28—C29	128.3 (2)
N1—C12—C1	116.45 (16)	C27—C28—S1	109.98 (16)
C10—C12—C1	101.57 (16)	C29—C28—S1	121.63 (16)
N1—C12—C13	103.05 (15)	C30—C29—C30'	94.7 (6)
C10—C12—C13	116.72 (16)	C30—C29—C28	131.1 (4)
C1—C12—C13	106.35 (15)	C30'—C29—C28	134.1 (4)
C17—C13—C14	114.85 (16)	C30'—C29—S2'	105.9 (4)
C17—C13—C12	111.36 (15)	C28—C29—S2'	120.0 (2)
C14—C13—C12	104.84 (16)	C30—C29—S2	106.4 (4)
C17—C13—H13	108.5	C28—C29—S2	122.32 (18)
C14—C13—H13	108.5	S2'—C29—S2	117.67 (19)
C12—C13—H13	108.5	C32—C31—C30	105.7 (4)
C25—C14—C15	114.28 (16)	C32—C31—S2'	119.4 (3)
C25—C14—C13	113.61 (17)	C32—C31—H31	127.1
C15—C14—C13	104.45 (16)	C30—C31—H31	127.1
C25—C14—H14	108.1	S2'—C31—H31	113.5
C15—C14—H14	108.1	C31—C32—C30'	103.6 (5)
C13—C14—H14	108.1	C31—C32—S2	118.3 (2)
N1—C15—C14	102.53 (16)	C31—C32—H32	120.9
N1—C15—H15A	111.3	C30'—C32—H32	135.5
C14—C15—H15A	111.3	S2—C32—H32	120.9
N1—C15—H15B	111.3	C12—N1—C16	115.98 (18)
C14—C15—H15B	111.3	C12—N1—C15	108.42 (15)
H15A—C15—H15B	109.2	C16—N1—C15	115.71 (17)
N1—C16—H16A	109.5	C28—S1—C25	92.78 (10)
N1—C16—H16B	109.5	C32—S2—C29	92.4 (2)
H16A—C16—H16B	109.5	C29—C30—C31	117.2 (7)
N1—C16—H16C	109.5	C29—C30—H30	121.4
H16A—C16—H16C	109.5	C31—C30—H30	121.4
H16B—C16—H16C	109.5	C31—S2'—C29	92.3 (3)
O2—C17—C18	121.07 (18)	C29—C30'—C32	118.8 (8)
O2—C17—C13	120.77 (19)	C29—C30'—H30'	120.6
C18—C17—C13	118.11 (18)	C32—C30'—H30'	120.6
			
O1—C1—C2—C3	10.4 (4)	C19—C20—C21—C24	−178.3 (2)
C12—C1—C2—C3	−171.0 (2)	C20—C21—C22—C23	−2.0 (3)
O1—C1—C2—C11	−168.1 (2)	C24—C21—C22—C23	177.3 (2)
C12—C1—C2—C11	10.5 (2)	C19—C18—C23—C22	0.6 (3)
C11—C2—C3—C4	1.4 (3)	C17—C18—C23—C22	−179.5 (2)
C1—C2—C3—C4	−176.9 (2)	C21—C22—C23—C18	1.2 (3)
C2—C3—C4—C5	1.6 (4)	C15—C14—C25—C26	−140.7 (2)
C3—C4—C5—C6	−2.2 (4)	C13—C14—C25—C26	−21.0 (3)
C4—C5—C6—C11	−0.2 (4)	C15—C14—C25—S1	41.9 (2)
C4—C5—C6—C7	−179.9 (3)	C13—C14—C25—S1	161.63 (14)
C11—C6—C7—C8	0.0 (4)	C14—C25—C26—C27	−177.1 (2)
C5—C6—C7—C8	179.7 (2)	S1—C25—C26—C27	0.5 (2)
C6—C7—C8—C9	−1.2 (4)	C25—C26—C27—C28	−0.1 (3)
C7—C8—C9—C10	0.5 (4)	C26—C27—C28—C29	177.0 (2)
C8—C9—C10—C11	1.3 (3)	C26—C27—C28—S1	−0.3 (3)
C8—C9—C10—C12	−178.0 (2)	C27—C28—C29—C30	16.4 (10)
C7—C6—C11—C2	−177.0 (2)	S1—C28—C29—C30	−166.6 (10)
C5—C6—C11—C2	3.3 (3)	C27—C28—C29—C30'	−157.7 (12)
C7—C6—C11—C10	1.9 (3)	S1—C28—C29—C30'	19.4 (12)
C5—C6—C11—C10	−177.8 (2)	C27—C28—C29—S2'	20.0 (4)
C3—C2—C11—C6	−3.9 (3)	S1—C28—C29—S2'	−162.9 (2)
C1—C2—C11—C6	174.77 (19)	C27—C28—C29—S2	−157.9 (3)
C3—C2—C11—C10	177.0 (2)	S1—C28—C29—S2	19.1 (3)
C1—C2—C11—C10	−4.3 (2)	C30—C31—C32—C30'	−2.4 (12)
C9—C10—C11—C6	−2.6 (3)	S2'—C31—C32—C30'	0.0 (9)
C12—C10—C11—C6	176.90 (19)	C30—C31—C32—S2	−2.0 (8)
C9—C10—C11—C2	176.44 (19)	S2'—C31—C32—S2	0.3 (5)
C12—C10—C11—C2	−4.1 (2)	C10—C12—N1—C16	−67.1 (2)
C9—C10—C12—N1	−45.2 (3)	C1—C12—N1—C16	50.0 (2)
C11—C10—C12—N1	135.43 (18)	C13—C12—N1—C16	166.00 (17)
C9—C10—C12—C1	−170.8 (2)	C10—C12—N1—C15	160.78 (17)
C11—C10—C12—C1	9.9 (2)	C1—C12—N1—C15	−82.1 (2)
C9—C10—C12—C13	74.1 (3)	C13—C12—N1—C15	33.87 (19)
C11—C10—C12—C13	−105.3 (2)	C14—C15—N1—C12	−42.9 (2)
O1—C1—C12—N1	43.1 (3)	C14—C15—N1—C16	−175.15 (19)
C2—C1—C12—N1	−135.53 (18)	C27—C28—S1—C25	0.51 (17)
O1—C1—C12—C10	166.4 (2)	C29—C28—S1—C25	−177.02 (18)
C2—C1—C12—C10	−12.2 (2)	C26—C25—S1—C28	−0.56 (17)
O1—C1—C12—C13	−71.0 (2)	C14—C25—S1—C28	177.31 (16)
C2—C1—C12—C13	110.34 (17)	C31—C32—S2—C29	0.7 (3)
N1—C12—C13—C17	113.41 (17)	C30'—C32—S2—C29	2(4)
C10—C12—C13—C17	−11.2 (2)	C30—C29—S2—C32	0.9 (8)
C1—C12—C13—C17	−123.60 (18)	C30'—C29—S2—C32	−3(5)
N1—C12—C13—C14	−11.37 (19)	C28—C29—S2—C32	176.4 (2)
C10—C12—C13—C14	−135.95 (17)	S2'—C29—S2—C32	−1.6 (3)
C1—C12—C13—C14	111.62 (17)	C30'—C29—C30—C31	−1.6 (15)
C17—C13—C14—C25	99.2 (2)	C28—C29—C30—C31	−177.3 (6)
C12—C13—C14—C25	−138.30 (17)	S2'—C29—C30—C31	167 (5)
C17—C13—C14—C15	−135.67 (18)	S2—C29—C30—C31	−2.3 (14)
C12—C13—C14—C15	−13.1 (2)	C32—C31—C30—C29	2.8 (14)
C25—C14—C15—N1	157.67 (17)	S2'—C31—C30—C29	−169 (5)
C13—C14—C15—N1	32.9 (2)	C32—C31—S2'—C29	−1.2 (4)
C14—C13—C17—O2	19.0 (3)	C30—C31—S2'—C29	8(3)
C12—C13—C17—O2	−100.0 (2)	C30—C29—S2'—C31	−10 (4)
C14—C13—C17—C18	−163.69 (17)	C30'—C29—S2'—C31	1.9 (9)
C12—C13—C17—C18	77.4 (2)	C28—C29—S2'—C31	−176.4 (2)
O2—C17—C18—C19	−154.7 (2)	S2—C29—S2'—C31	1.7 (3)
C13—C17—C18—C19	27.9 (3)	C30—C29—C30'—C32	−0.1 (16)
O2—C17—C18—C23	25.3 (3)	C28—C29—C30'—C32	175.4 (7)
C13—C17—C18—C23	−152.00 (19)	S2'—C29—C30'—C32	−2.5 (17)
C23—C18—C19—C20	−1.6 (3)	S2—C29—C30'—C32	177 (6)
C17—C18—C19—C20	178.5 (2)	C31—C32—C30'—C29	1.7 (17)
C18—C19—C20—C21	0.8 (4)	S2—C32—C30'—C29	−177 (5)
C19—C20—C21—C22	1.0 (4)		

### Hydrogen-bond geometry (Å, °)

**Table:**

Cg8 is the centroid of the C18–C23 ring.

**Table:**

*D*—H···*A*	*D*—H	H···*A*	*D*···*A*	*D*—H···*A*
C32—H32···Cg8^i^	0.93	2.86	3.661 (3)	145

Symmetry codes: (i) −*x*−1/2, *y*+1/2, −*z*+3/2.
